# Understanding the molecular basis of resilience to Alzheimer’s disease

**DOI:** 10.3389/fnins.2023.1311157

**Published:** 2023-12-19

**Authors:** Kathleen S. Montine, Eloïse Berson, Thanaphong Phongpreecha, Zhi Huang, Nima Aghaeepour, James Y. Zou, Michael J. MacCoss, Thomas J. Montine

**Affiliations:** ^1^Department of Pathology, Stanford University, Stanford, CA, United States; ^2^Department of Anesthesiology, Stanford University, Stanford, CA, United States; ^3^Department of Biomedical Data Science, Stanford University, Stanford, CA, United States; ^4^Department of Computer Science, Stanford University, Stanford, CA, United States; ^5^Department of Genome Sciences, University of Washington, Seattle, WA, United States

**Keywords:** aging, cognition, computational models, dementia, proteomic analysis, neuropathologic lesion, machine learning

## Abstract

The cellular and molecular distinction between brain aging and neurodegenerative disease begins to blur in the oldest old. Approximately 15–25% of observations in humans do not fit predicted clinical manifestations, likely the result of suppressed damage despite usually adequate stressors and of resilience, the suppression of neurological dysfunction despite usually adequate degeneration. Factors during life may predict the clinico-pathologic state of resilience: cardiovascular health and mental health, more so than educational attainment, are predictive of a continuous measure of resilience to Alzheimer’s disease (AD) and AD-related dementias (ADRDs). In resilience to AD alone (RAD), core features include synaptic and axonal processes, especially in the hippocampus. Future focus on larger and more diverse cohorts and additional regions offer emerging opportunities to understand this counterforce to neurodegeneration. The focus of this review is the molecular basis of resilience to AD.

## 1 Introduction

Neurodegenerative diseases span from newborns to centenarians and from ultra-rare to highly prevalent. The more prevalent neurodegenerative diseases are an emerging pandemic as the world’s age demographic historically shifts to older age because of success in treating acute diseases, and underscores the growing need to manage chronic diseases. Although age-related neuropathologies account for a large proportion of late life cognitive decline, considerable variation remains unexplained even after considering a wide array of neuropathologies (Boyle et al., 2021). Here we will focus on the most prevalent age-related neurodegenerative disease, Alzheimer’s disease (AD), and its commonly comorbid conspirators, the so-called AD-related dementias (ADRDs).

**Figure 1:**
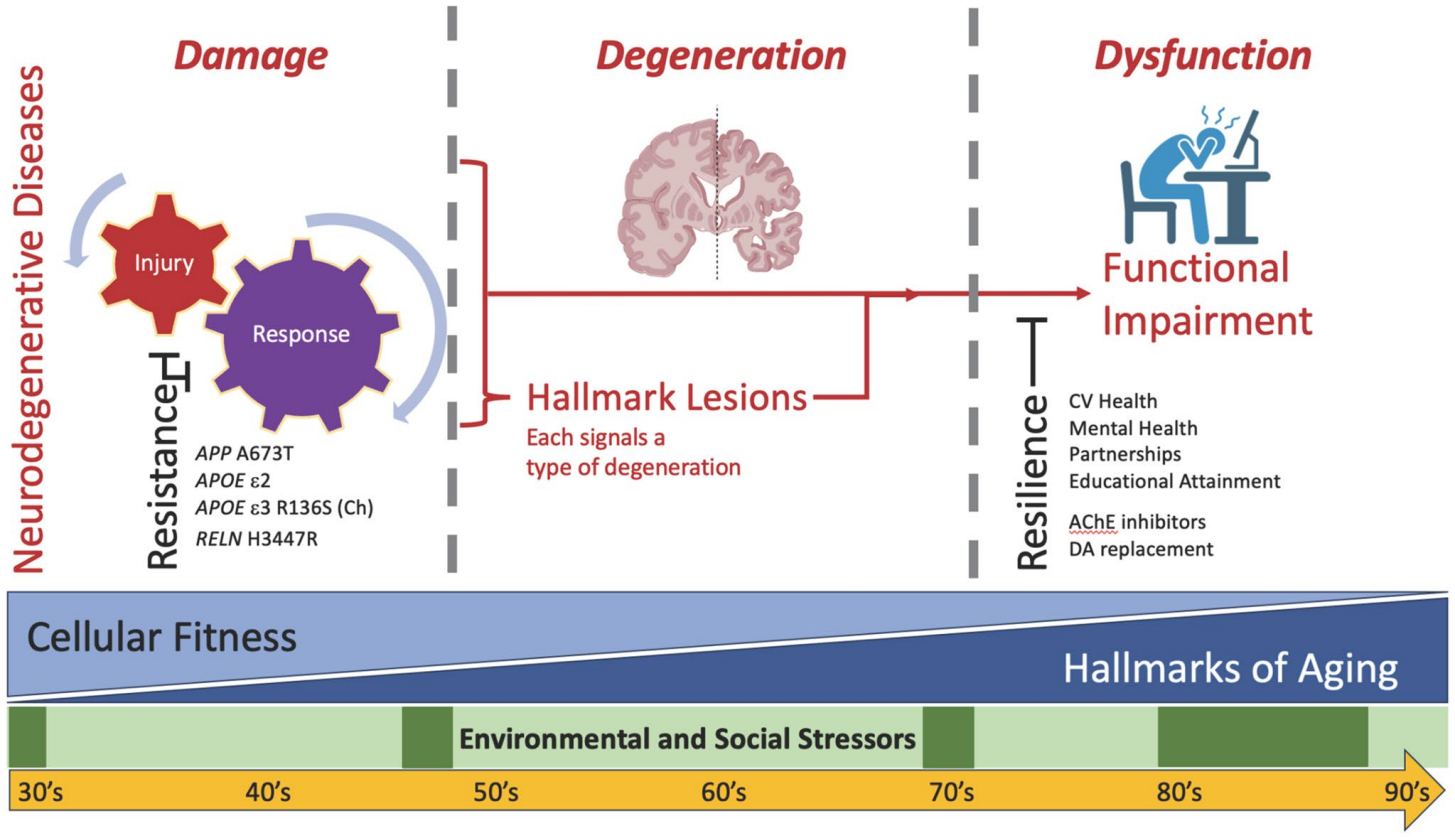
Diagram of the hypothetical relationships among environmental and social stressors, brain aging, and neurodegenerative disease(s). While time passes for all of us, two sources of cellular and molecular stress occur in everyone’s brain to varying extents: episodic environmental and social stressors (layer 1, green) and the biology of aging with attendant reduced fitness for all cell types in brain (layer 2, blue). Biology of aging progressively amplifies with advancing age, although at varying rates across individuals and across organs within an individual. On their own, mounting stressors from these two layers appear insufficient to cause neurodegenerative disease(s). Addition of a third source (layer 3, red)—the neurodegenerative etiologic factors underlying injury plus response to injury—initiates additional cellular and molecular damage, leading to degeneration as signaled by disease-specific formation of hallmark pathologic lesions, including amyloid beta plaques and tau tangles for AD. It is important to recognize that damage may contribute to degeneration through pathways that are dependent or independent of hallmark lesion formation. Sufficiently severe degeneration ultimately expresses as clinically detectable dysfunction. Because this direct model of damage to degeneration to dysfunction is insufficient to account for all observations in humans and experimental animals, two impedance terms are required: resistance and resilience. Resistance signifies suppressed damage despite usually adequate stressors (left), and resilience signifies suppressed functional impairment (right) despite usually adequate degeneration. Examples are provided for some resistance and resilience factors; see text for details.

During our lifetimes, we varyingly experience episodic social and environmental stressors, some of which appear epidemiologically to have a major impact on the risk of disease, including AD and ADRDs (Hunt et al., 2020; Livingston et al., 2020; Besser, 2021; Peterson et al., 2021; Zhao et al., 2021; Chen et al., 2022). This is the first layer of often episodic stressors that impact the brain (Figure 1, green) and include life events such as social isolation and death of a spouse (Wu-Chung et al., 2022). The molecular mechanisms by which these social and environmental stressors influence the subsequent development of neurodegeneration are not yet clear but are an active area of investigation.

An influential perspective on the cellular and molecular hallmarks of aging published a decade ago (López-Otín et al., 2013), and recently updated (López-Otín et al., 2023), highlights key features of the biology of aging. Importantly, age—the time lived by an organism—is an imperfect proxy for the underlying biology of aging. Indeed, the cellular and molecular hallmarks of aging accumulate at different rates in different species, in different individuals within a species, and in different organs within an individual. With cancer as the one exception, their net effect is to decrease cellular fitness and thereby promote age-related diseases: this is the second layer of stressors experienced by the aging brain (Figure 1, blue). Importantly, the majority of the hallmarks of aging are shared with the hallmarks of neurodegenerative diseases, including neuropathologic lesions such as amyloid beta plaques and tau tangles (Gómez-Isla and Frosch, 2022; Wilson et al., 2023). Indeed, blinded research neuropathologists would not be expected to distinguish who had dementia from who was cognitively unimpaired purely from histologic analysis of the brain, qualitatively blurring the cellular and molecular features of aging versus neurodegenerative diseases, especially in the oldest old (Montine et al., 2022). Also an active area of investigation, Mathys et al. recently generated a single-cell transcriptomic atlas to assess varying degrees of AD pathology and cognitive impairment and found neuronal (DNA damage) and microglial (genetic variation) differences between aging and neurodegeneration (Mathys et al., 2023).

Because these usual social and environmental influencers and inevitably diminished cellular fitness from aging appears to be insufficient to cause neurodegenerative disease, a third layer of stressors, etiologic determinants of neurodegenerative disease(s) (Wilson et al., 2023), appears to be required before clinical dysfunction becomes apparent (Figure 1, red). In this “direct model,” these three layers of stressors are required to drive enough damage to lead to sufficient degeneration to result in observable dysfunction. To model the real-world situation, a very common experimental paradigm is to create genetically engineered mice that drive a particular type of injury (layer 3) and then age these animals (layer 2) to introduce diminished cellular fitness that together promote degeneration that leads to dysfunction, all while maintaining tight control of the environment (layer 1). Interestingly, many groups have shown the profound impact of environment, reporting that alterations in genetic background or strain of mouse, or even changing diet in the same strain of mouse, can markedly suppress degeneration and its downstream behavioral consequences in these types of experiments despite the continued strong expression of the genetic alteration (Karsten and Geschwind, 2005; Mattson et al., 2017).

Such data from mice challenge the sufficiency of the direct model where damage to degeneration results in dysfunction and require introducing an impedance term for damage, which we refer to as resistance (Figure 1, left), meaning resistance to damage and its downstream consequences of hallmark lesions of degeneration, despite continued application of stressor(s) (Latimer et al., 2017; Montine et al., 2019; Biswas et al., 2023). In mice, one of the best- known examples of resistance is removal of *apoE* in a mouse model genetically engineered to overexpress human amyloid beta; this was shown 25 years ago to suppress dramatically the accumulation of human amyloid beta plaques (Bales et al., 1997). Resistance factors, although relatively uncommon, have been identified in humans. Continuing with examples from AD, the *APP* A673T variant suppresses amyloid beta production (Di Fede et al., 2009; Jonsson et al., 2012), while *APOE* R136S (Christchurch) and *RELN* H3447R resist downstream pathologic tau accumulation (Arboleda-Velasquez et al., 2019; Lopera et al., 2023).

Similar to resistance, a second impedance term—resilience—is commonly used to describe a mismatch between degeneration resulting from damage and predicted dysfunction (Figure 1, right). Initially observed in community-based cohorts, but now reported in virtually every research cohort that includes older people systematically assessed, somewhere between 10 to 30% of individuals (estimates vary by average age and the definitions used) harbor a high burden of degeneration sufficient to diagnose dementia in others, yet remain cognitively normal or even high cognitive performers (reviewed in Gómez-Isla and Frosch, 2022). These observations reveal another insufficiency in the direct model, at least in humans, requiring the introduction resilience, to describe the gap between neurodegeneration and expected clinical expression of cognitive impairment (Hohman et al., 2016; Montine et al., 2019; Melikyan et al., 2022). The idea of resilience is familiar to the field neurodegenerative disease, with drugs for both Parkinson’s disease (dopamine replacement therapy) and AD (acetylcholinesterase inhibitors) that promote resilience. AD resilience is estimated to be up to 10-times more common than resistance, and resilience to each of the ADRDs also has been reported (Montine et al., 2022; Walker and Richardson, 2023; Willroth et al., 2023). Here we review work our group has done to predict resilience and identify its molecular features.

## 2 Predicting resilience during life

Recently we defined cognitive resilience (CR) as a continuum instead of the traditional binary (present or absent) categorization, allowing a more comprehensive assessment of resilience and its relationship to other cognitive variables, and overcoming the drawbacks of binary categorization (Phongpreecha et al., 2023b). Indeed, binary categorization of CR captures only the most extreme examples and neglects its likely variable expression by different people and across different cognitive domains. To operationalize CR, we used our previously described (Phongpreecha et al., 2023a) neuropathologic damage estimate derived using a machine learning (ML) approach that combined 17 lesions (including plaques, tangles, LBs, hippocampal sclerosis, and TDP-43) into a single index, along with assessments of cognitive function as determined by neuropsychological tests or clinical assessment. Our model simplifies the relationship into linear equations:


(1) Cognitive measure=CRScores−Damage Estimate

Where cognitive measure is a result of one’s cognitive resilience (CR) minus the damage incurred from diseases such as AD and ADRDs. Thus, CR Score can be solved using quantifiable cognitive measures and damage estimates, as our ML model converts the Damage Estimate into the same unit as cognitive measures, allowing the calculation of CR Scores for different cognitive tests (Phongpreecha et al., 2023b). We conceptualized CR to derive from two components (Eq. 2):


(2) CRScore=Reserve+Compensation

Where reserve represents capacity built up over the lifetime prior to the onset of disease (premorbid) and compensation represents adaptation of existing capacity in response to disease (morbid), although we cannot as yet measure their relative contributions, which occur at different times in life. Reserve accumulates prior to disease onset, such as maximizing bone calcium content in youth, while compensation occurs in response to disease and so follows neuropathologic damage, such as recruitment of additional regions of the brain to subserve memory function. Increased reserve may underlie previous observations that more extensive neuropathology is found in younger AD cases (Morgan, 1992), consistent with protection from clinical manifestation until their greater reserve capacity was depleted.

**Figure 2:**
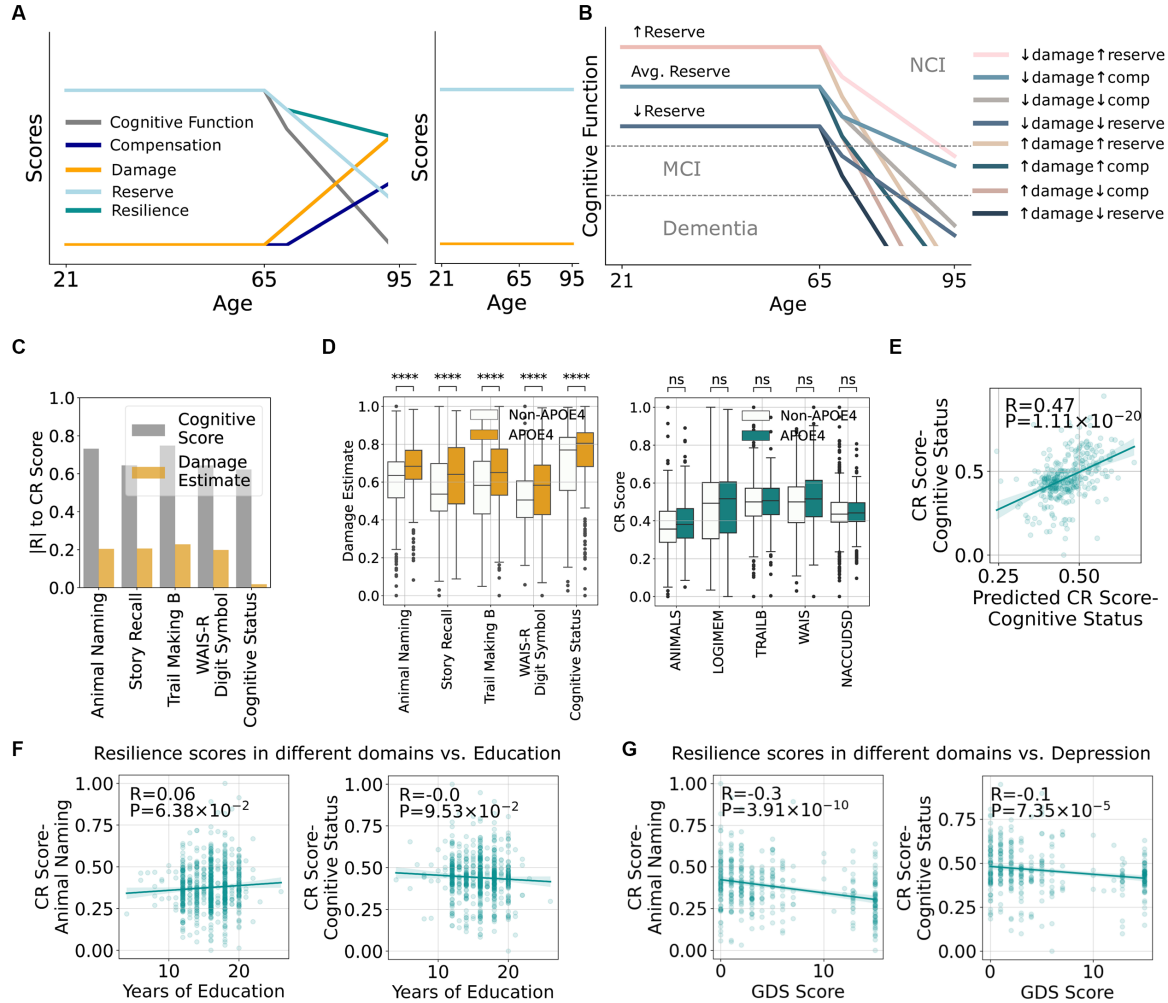
A framework that quantitatively defines resilience and its relationship to other cognitive terms suggest minimal overlap between mechanisms underlying damage and resilience. **(A)** Hypothetical relationships between increasing damage as an individual ages and how it impacts existing cognitive reserve, resilience, compensation, and cognitive function (left graph). The right graph shows an example of resistance with minimal damage across the lifespan and therefore no reduction in cognitive function. **(B)** Potential scenarios of cognitive impairment depending on the different quantities of damage, compensation, and reserve. **(C)** Correlations between estimated damage and cognitive resilience (CR score) from Eq. (1) stratified by cognitive tests. **(D)** Correlations between presence of *APOE* ε4 and damage or CR score stratified by cognitive tests. **(E)** Actual CR scores vs. CR scores predicted by clinical features. **(F)** Correlation between CR scores from number of story recall or cognitive status with educational attainment. **(G)** Correlation between CR scores from number of story recall or cognitive status with geriatric depression score (GDS).

Although simplistic, these equations provide a framework for the impact of varying resilience on related terms. Visualizing the interactions among these terms as one ages shows different cognitive trajectories in different situations. In Figure 2A, reserve is largely unused up to middle age (Stern, 2002; Stern, 2012). Later, if an individual suffers progressively more damage to the brain from AD and ADRDs, cognitive function will decrease as will CR, a balance between drawing down reserves and, following a short delay, launching compensatory processes (Montine et al., 2019). Figure 2B shows that individuals with higher reserve have a greater premorbid capacity to offset damage and are more likely to preserve cognitive function into older ages, an outcome supported by clinical studies showing higher baseline cognitive function is associated with reserve (Hayden et al., 2019; Beker et al., 2020; Pettigrew et al., 2023). In this scenario, resistance represents a special case where there is none-to-minimal damage (Latimer et al., 2017; Montine et al., 2022) and hence no need to draw on reserve or compensation, (Figure 2A right).

While CR Scores show moderate correlations to multiple cognitive measures, including cognitive diagnosis, this is not the case for Damage Estimate (Figure 2C). This implies that the mechanisms underlying damage and resilience may be largely independent, i.e., being resilient does not meaningfully impact the amount of damage. In addition, *APOE* ε4 is a well-established risk factor for AD and some ADRDs, and Figure 2D confirms that the presence of *APOE* ε4 leads to a larger Damage Estimate in all cognitive measures. Surprisingly, *APOE* ε4 does not lead to lower CR Scores as described by previous studies using binary resilience concepts (Legdeur et al., 2018; Snitz et al., 2020; Wang et al., 2022) again suggesting potentially different pathways underlying damage and resilience. These results highlight the value of defining resilience quantitatively and decoupling its effects from damage as assessed by neuropathologic lesions.

With CR defined by clinico-pathological comparison, we next tested the hypothesis that CR Score might be predicted by features during life without using post-mortem data. For example, Figure 2E shows a moderate prediction performance of CR Score using a random forest model based on *intra vitam* features alone. Educational attainment has been so widely associated with resilience that it is sometimes used as a synonymous measure (Boots et al., 2015; Dekhtyar et al., 2019; Vonk et al., 2019; Fratiglioni et al., 2020; Ossenkoppele et al., 2020). While our study validated that educational attainment is significantly associated with CR Score as measured by some cognitive assessments (Figure 2F), the effect size was small. Other features that were used in the model, such as cardiovascular health and depression scores (Figure 2G), are more strongly correlated with CR Scores. This prediction opens venues for future research to determine genetic, medical, social, and environmental determinants of resilience and develop interventions to enhance resilience.

## 3 Molecular features of resilience to AD

We have also begun to define the molecular features of categorically-defined examples of resilience to high level AD. Here, we used a relatively small set of 43 highly selected brain donations from sex- and age-matched individuals who met stringent clinico-pathologic criteria for three groups: healthy control (HC), AD dementia (ADD), and resilience to AD (RAD), where all groups were free of common co-morbid pathologic changes. We then applied both unbiased data-independent peptide-based proteomics and bulk ATAQ-seq followed by cell-type deconvolution using a novel application called Cellformer to four regions of brain: superior and middle temporal gyri, inferior parietal lobule, hippocampus, and caudate nucleus.

**Figure 3:**
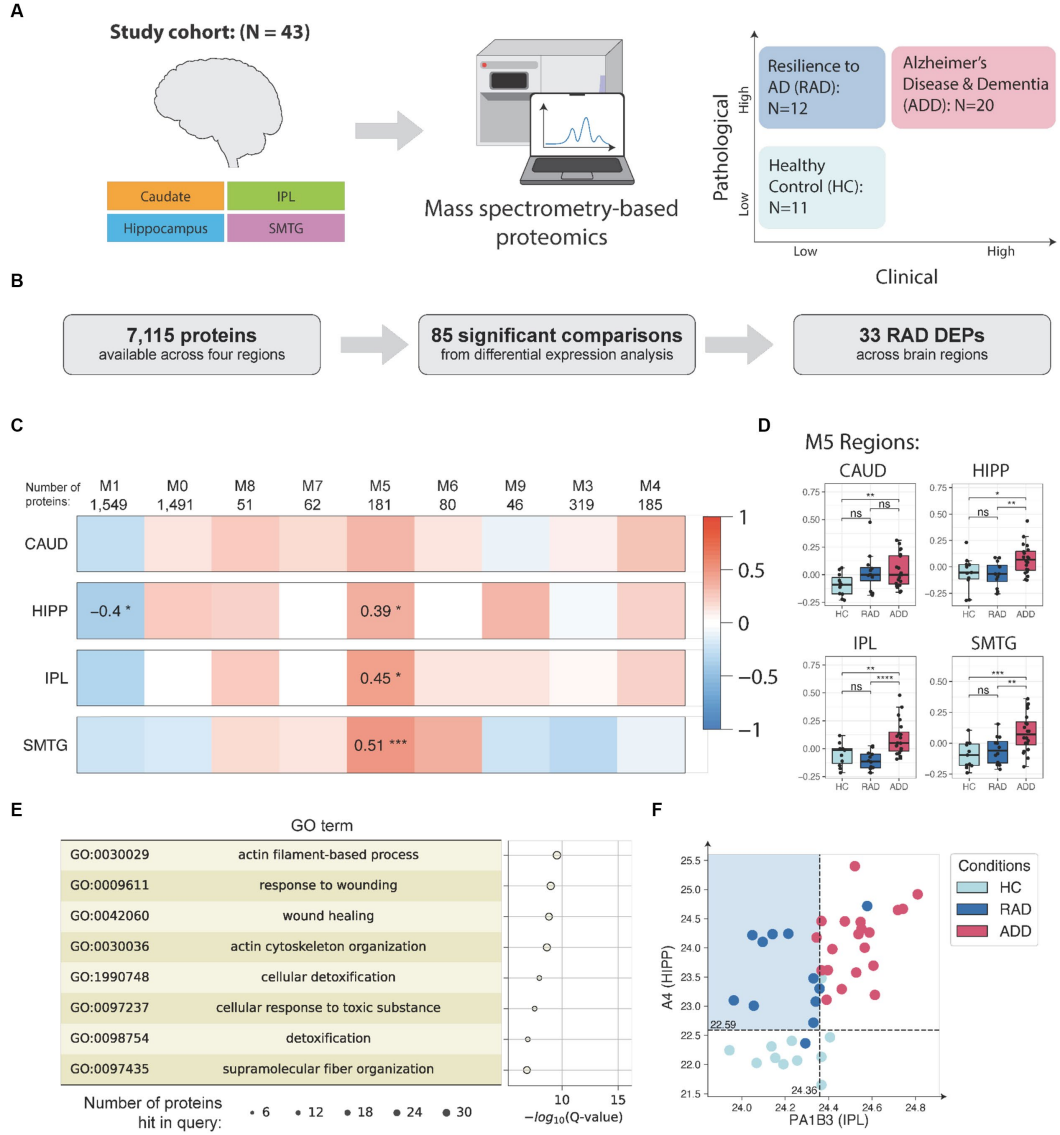
**(A)** Samples (*N* = 155) from up to four matched brain regions were donated by 43 research participants who were assigned to three clinico-pathologic groups: healthy control (HC), cognitive resilience to AD (RAD), or AD dementia (ADD). Samples were quantified by data independent tandem mass spectrometry and data analyzed by differential expression and co-expression network analyses. **(B)** Illustration of differential expression analysis and summary of the final number of RAD-associated differentially expressed proteins (RAD DEPs). **(C)** Consensus protein co-expression analysis identified 9 modules across four brain regions. Pearson correlation with two-sided *p*-values was used to evaluate the relationships between clinico-pathologic groups and eigenprotein expression. **(D)** Module 5 (M5) eigenprotein expressions in HC, RAD, and ADD for the study set. For the boxplots, the interior horizontal line represents the median value, the upper and lower box edges represent the 75th and 25th percentile, and the upper and lower bars represent the 90th and 10th percentiles, respectively. **(E)** Top enriched GO biological process terms in M5 and their enrichment analysis and the corresponding Q-values (with FDR B&H method) and number of proteins hit in query. **(F)** Using Aβ abundance in HIPP and PA1B3 concentration in IPL to distinguish RAD from other groups. The number of samples in the study cohort: HC = 11, RES = 12, ADD = 20. CAUD, caudate; HIPP, hippocampus; IPL, inferior parietal lobule; SMTG, superior and middle temporal gyrus; HC, healthy control; RAD, resilience to AD; ADD, AD and dementia; DLPFC, dorsolateral prefrontal cortex; PC, precuneus; GO, gene ontology. ^*^p < 0.05; ^**^p < 0.01; ^***^p < 0.001; ^****^p < 0.0001; ns, not significant.

Our approach to the proteomic work is summarized in Figure 3A and Huang et al. (2023a). The study identified 33 differentially expressed proteins across four brain regions that are associated with RAD (Figure 3B). One of the key findings of this study was the identification of lower levels of soluble amyloid beta (Aβ) in isocortical and hippocampal regions in individuals exhibiting RAD compared to ADD. This suggests that reducing soluble Aβ concentration in tissue, despite high level neuropathologic change, could play a pivotal role in mitigating cognitive impairment.

Further analysis with protein co-expression analysis revealed module #5 of 181 densely interacting proteins significantly associated with RAD (Figure 3C). Module #5 contains 14 out of the 33 differentially expressed proteins, including C04A, C04B, CAPG, HSPB1, K2C7, K2C8, CLUS, GFAP, FAAA, PRDX1, PA1B3, CMBL, ICAM1, and IRGQ. Moreover, the module #5 eigenprotein is able to distinguish RAD from ADD across HIPP, IPL, and SMTG regions (Figure 3D). Further gene ontology (GO) analysis suggests that these proteins are enriched for processes related to actin filament-based processes, cellular detoxification, and wound healing (Figure 3E). In addition, a decision tree classifier (Huang et al., 2023b) allows us to identify the top two protein features, Aβ in HIPP and PA1B3 in IPL, that were best at distinguishing the three clinico-pathological groups (Figure 3F). These proteomic findings were validated using data from four independent external cohorts, comprising a total of 689 human isocortical samples. This comprehensive study provides insights into the protein features of RAD.

**Figure 4:**
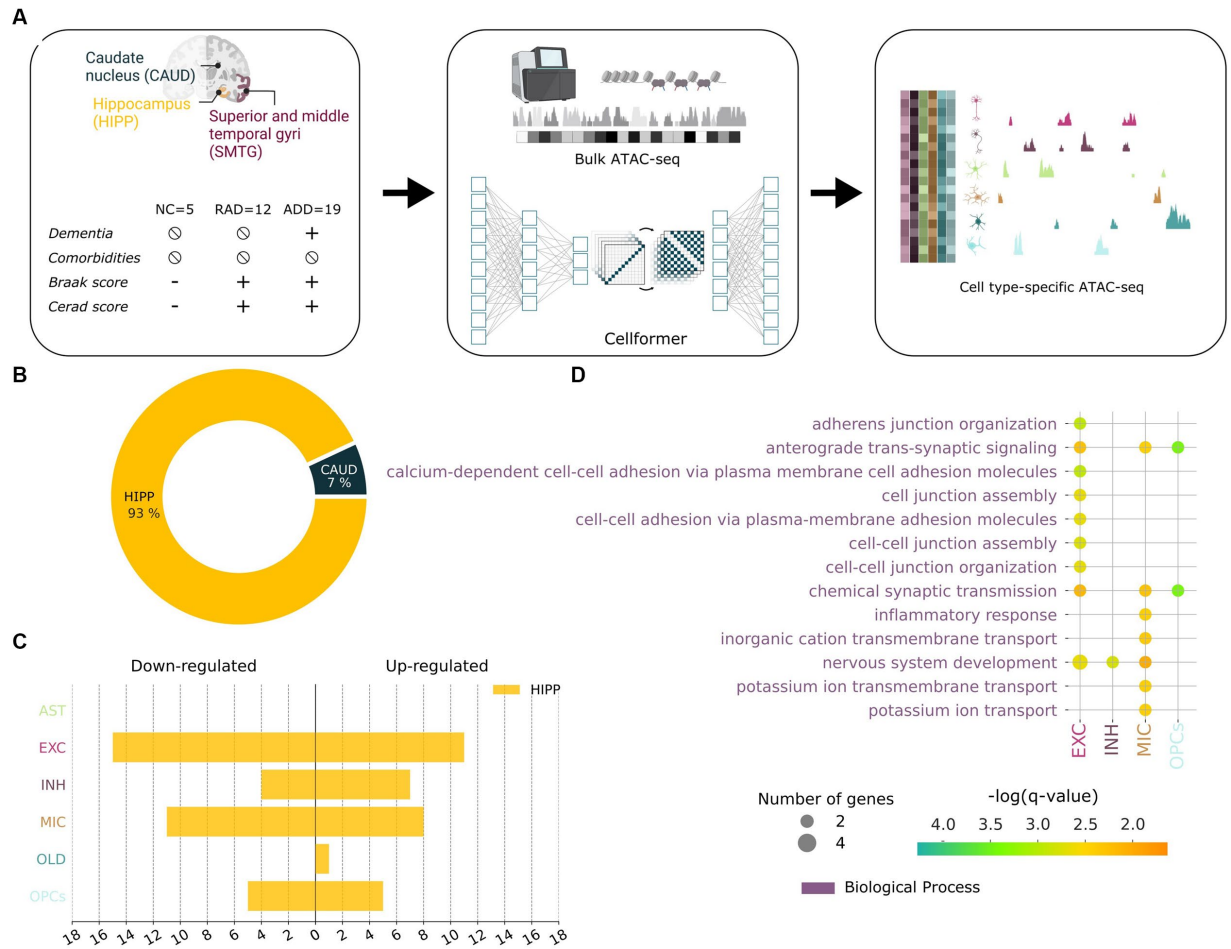
Cellformer deconvolutes epigenetic bulk expression into cell type–specific expression enabling an unprecedented open chromatin profiling of RAD. **(A)** Cellformer was fed data from comorbidity-free bulk samples from individuals with clinico-pathologic characterization as normal control (NC), Resilient to AD (RAD), and AD dementia (ADD). Three brain regions were used per individual to gain insight into the regional and cellular epigenetic profile of RAD. Cellformer generated cell type–specific expression for 6 main cell types across the whole genome, leading to an unprecedented chromatin profiling of RAD. **(B)** Cell type–specific open chromatin region (OCR) between RAD and ADD/NC were mainly found in HIPP (93%) and distributed between microglia (28%), and neuron cells (55%) (adjusted *p* < 0.05, logFC >0.5). **(C)** Number of OCRs (x-axis) differentially upregulated and downregulated in RAD compared to ADD/NC across cell types (y-axis). **(D)** GO enrichment applied to RAD-specific OCR (FDR 5%).

Leveraging 191 well-curated tissue samples from the same clinico-pathologic groups—Healthy Control (HC, *n* = 5), RAD (*n* = 12), and ADD (*n* = 19) from the same cohort (Berson et al., 2023)—we investigated epigenetic features underpinning RAD in three brain regions SMTG, hippocampus, and caudate (Figure 4A). We used bulk ATAC-seq data coupled with Cellformer, a deep learning algorithm that deconvolutes cell type–specific open chromatin region (OCR) accessibility from bulk data, to provide a comprehensive understanding of heterogeneity in chromatin accessibility across cell populations and cell-specific OCR variation in different brain regions of people with categorically defined RAD.

Cellformer predicted that OCR differences between RAD and the other two groups that are on the AD continuum are very strongly localized to HIPP, which subserves declarative memory formation and is the primary target of AD (Figure 4B). At the cellular level, most predicted RAD-specific OCR were characterized by changes in both inhibitory and excitatory neurons followed by microglia (Duggan and Parikh, 2021; Figure 4C). From the perspective of cellular processes, GO analysis of RAD-specific OCR highlighted neuronal development, inflammatory response, and synaptic transmission processes (Figure 4D). These pathways were highlighted in previous studies using proteomics and mouse models of AD (Kaczorowski et al., 2011; Arnold et al., 2013; Seto et al., 2021; Neuner et al., 2022). Overall these highly plausible predictions suggest that individuals with RAD are distinguished from the AD continuum by epigenetic upregulation in support of hippocampal neuronal processes and synapses. This difference in the regulome might confer RAD the ability to preserve the number of neuronal projections and synapses that has been observed through histopathological studies (Perez-Nievas et al., 2013).

## 4 Discussion

Episodic social and environmental stressors combine with the progressively amplifying biology of aging to reduce cellular fitness in brain as we age. Although on their own, these two stressors of the aging brain are insufficient to cause neurodegenerative disease, the cellular and molecular distinction between brain aging and neurodegenerative disease begins to blur in the oldest old. Usually, a third stressor, the etiologic factors that drive damage from neurodegenerative disease(s), conspires with social/environmental influences and processes of aging to yield cellular and molecular damage that leads to degeneration with hallmark pathologic lesions that lead to neurological dysfunction. Approximately 15 to 25% of observations in humans do not fit this hypothetical direct model of damage to degeneration to dysfunction, requiring the introduction of two impedance terms: resistance, which suppress damage despite usually adequate stressors, and the more common resilience, which suppress neurological dysfunction despite usually adequate degeneration. Here, we reviewed recent work to determine factors during life that predict the clinico-pathologic state of resilience and showed that cardiovascular health and mental health, more so than educational attainment, are predictive of a continuous measure of resilience to AD and ADRDs. We next focused on molecular features of RAD—resilience to AD alone—and observed that synaptic and axonal processes, especially in the hippocampus, are core features of RAD. Future work may focus on larger and more diverse cohorts that include ADRDs and explore additional regions of brain for their contribution to this counterforce to neurodegeneration.

## Author contributions

KM: Project administration, Writing – original draft, Writing – review & editing. EB: Visualization, Writing – original draft, Writing – review & editing. TP: Visualization, Writing – original draft, Writing – review & editing. ZH: Visualization, Writing – original draft, Writing – review & editing. NA: Funding acquisition, Resources, Supervision, Writing – review & editing. JZ: Funding acquisition, Resources, Supervision, Writing – review & editing. MM: Funding acquisition, Resources, Writing – review & editing. TM: Conceptualization, Funding acquisition, Supervision, Writing – original draft, Writing – review & editing.
